# Diseases of Poverty and Lifestyle, Well-Being and Human Development

**DOI:** 10.4103/0973-1229.40567

**Published:** 2008

**Authors:** Ajai R. Singh, Shakuntala A. Singh

**Affiliations:** * Psychiatrist. Editor, Mens Sana Monographs, India; ** Reader and Head, Dept. of Philosophy, Joshi-Bedekar College, Thane, India. Deputy Editor, Mens Sana Monographs, India

**Keywords:** *Diseases of Poverty*, *Lifestyle Diseases*, *Optimism Deprivation*, *Farmer Suicides*, *Capability Deprivation*, *Well-Being*, *Longevity*, *Professional Burnout*, *Psychosomatic Ailments*, *Human Development*, *Faulty Lifestyle*, *Lifestyle Stress*, *Health Promoting Behaviours*, *Negative Emotions*, *Positive Health*, *The Simplicity Movement*

## Abstract

The problems of the haves differ substantially from those of the have-nots. Individuals in developing societies have to fight mainly against infectious and communicable diseases, while in the developed world the battles are mainly against lifestyle diseases. Yet, at a very fundamental level, the problems are the same-the fight is against distress, disability, and premature death; against human exploitation and for human development and self-actualisation; against the callousness to critical concerns in regimes and scientific power centres.

While there has been great progress in the treatment of individual diseases, human pathology continues to increase. Sicknesses are not decreasing in number, they are only changing in type.

The primary diseases of poverty like TB, malaria, and HIV/AIDS-and the often co-morbid and ubiquitous malnutrition-take their toll on helpless populations in developing countries. Poverty is not just income deprivation but capability deprivation and optimism deprivation as well.

While life expectancy may have increased in the haves, and infant and maternal mortality reduced, these gains have not necessarily ensured that well-being results. There are ever-multiplying numbers of individuals whose well-being is compromised due to lifestyle diseases. These diseases are the result of faulty lifestyles and the consequent crippling stress. But it serves no one's purpose to understand them as such. So, the prescription pad continues to prevail over lifestyle-change counselling or research.

The struggle to achieve well-being and positive health, to ensure longevity, to combat lifestyle stress and professional burnout, and to reduce psychosomatic ailments continues unabated, with hardly an end in sight.

We thus realise that morbidity, disability, and death assail all three societies: the ones with infectious diseases, the ones with diseases of poverty, and the ones with lifestyle diseases. If it is bacteria in their various forms that are the culprit in infectious diseases, it is poverty/deprivation in its various manifestations that is the culprit in poverty-related diseases, and it is lifestyle stress in its various avatars that is the culprit in lifestyle diseases. It is as though poverty and lifestyle stress have become the modern “bacteria” of developing and developed societies, respectively.

For those societies afflicted with diseases of poverty, of course, the prime concern is to escape from the deadly grip of poverty-disease-deprivation-helplessness; but, while so doing, they must be careful not to land in the lap of lifestyle diseases. For the haves, the need is to seek well-being, positive health, and inner rootedness; to ask science not only to give them new pills for new ills, but to define and study how negative emotions hamper health and how positive ones promote it; to find out what is inner peace, what is the connection between spirituality and health, what is well-being, what is self-actualisation, what prevents disease, what leads to longevity, how simplicity impacts health, what attitudes help cope with chronic sicknesses, how sicknesses can be reversed (not just treated), etc. Studies on well-being, longevity, and simplicity need the concerted attention of researchers.

The task ahead is cut out for each one of us: physician, patient, caregiver, biomedical researcher, writer/journalist, science administrator, policy maker, ethicist, man of religion, practitioner of alternate/complementary medicine, citizen of a world community, etc. Each one must do his or her bit to ensure freedom from disease and achieve well-being.

Those in the developed world have the means to make life meaningful but, often, have lost the meaning of life itself; those in the developing world are fighting for survival but, often, have recipes to make life meaningful. This is especially true of a society like India, which is rapidly emerging from its underdeveloped status. It is an ancient civilization, with a philosophical outlook based on a robust mix of the temporal and the spiritual, with vibrant indigenous biomedical and related disciplines, for example, Ayurveda, Yoga, etc. It also has a burgeoning corpus of modern biomedical knowledge in active conversation with the rest of the world. It should be especially careful that, while it does not negate the fruits of economic development and scientific/biomedical advance that seem to beckon it in this century, it does not also forget the values that have added meaning and purpose to life; values that the ancients bequeathed it, drawn from their experiential knowledge down the centuries.

The means that the developed have could combine with the recipes to make them meaningful that the developing have. That is the challenge ahead for mankind as it gropes its way out of poverty, disease, despair, alienation, anomie, and the ubiquitous all-devouring lifestyle stresses, and takes halting steps towards well-being and the glory of human development.

## Introduction

Biomedicine has to engage in battles on numerous fronts. While individual diseases have to be tackled, patient welfare safeguarded, and scientific progress forwarded, it also has to address the social forces that impinge on, regulate, modify, and at times derail many an earnest effort at disease control. Socioeconomic and political factors, along with public awareness, are three crucial areas that cannot be neglected if the fight against disease and for positive health, well-being, and human development has to succeed. The main culprits here are poverty in the have-nots and lifestyle stresses in the haves, and both are interlinked with callousness in those who have the power to change things.

The problems of the haves differ substantially from those of the have-nots. Their concerns are different, as are their diseases. The social issues, interpersonal problems, and cultural ethos in the two groups are markedly different. Yet, at a very fundamental level, their problems remain the same–both fight against distress, disability, and premature death; they struggle against human exploitation and for human development and self-actualisation; and they struggle against callousness to critical concerns in regimes and scientific power centres. The haves are not any better off than the have-nots on these parameters, although they may appear to be so. It is only that the issues of disease, well-being, development, and the fight against callousness adopt different forms in these two groups. We will see later how this is true.

Also worth noting is the fact that the number of people falling sick has not reduced. While individual disease treatment is progressing, so also is human pathology; sicknesses are not reducing in number; they are only changing in type (Singh and Singh, 2005-2006):

Health awareness has increased. So has average life expectancy. Medical science boasts of a vast array of treatment modalities for an equally vast array of diseases. Distress has been ameliorated, disability curtailed, death postponed.

And yet, if the booming medical practice and pharma industry are any indication, the patient population has not reduced. In fact, it has multiplied. Not all of this is because of increased health awareness. While individual distress may have been reduced, individual disability curtailed, and individual death postponed due to better treatment facilities, the number of distressed have not reduced. Neither have the number of disabled, nor that of the dead.

What does this signify?

It signifies, if nothing else, that while individual disease treatment is progressing, so also is human pathology. Newer and more ingenious ways of falling ill are seeing the light of day, and the body is finding newer ways of getting out of order.

Sicknesses are not reducing in number. They are changing in type. If infectious diseases and malnutrition took their toll in the earlier centuries (and in certain sections of the world even today), lifestyle diseases, chronic conditions, and neoplastic disorders are taking their toll in the present. It is almost like changing fashions in the world of disease (Singh and Singh, 2005-2006).

Hence, it indeed is an unrelenting struggle to keep disease at bay and ensure human development and well-being. In all such struggles, both in the haves and the have-nots, people from all strata of society-high, middle, or low-and in different types of societies-Asian, European, African, American, or Australian-are perennially involved. Those who consider themselves immune to such considerations only cloak their ignorance in false bravado.

Let us see first the problems of the have-nots, then of the haves, and then of societies and people in transition. We could then analyse the essential factors that impact all three, their commonalities and differences, and what could be the action plan to meet them head-on.

## The Problem of the Have-Nots

Primary diseases of poverty like TB, malaria, and HIV/AIDS-and often the co-morbid and ubiquitous malnutrition-take their toll in helpless populations in developing countries. Note, for example:

1. Nearly 95% of almost 40 million victims of AIDS live in developing countries, with over 28 million in Africa alone (UNFPA State of World Population, 2007a). Its impact is greatest among the poor, who have no economic cushion and the weakest social support of any group; it is the leading cause of death in sub-Saharan Africa and the world's fourth biggest killer (ibid).
2. 98% of the world's active TB cases are in developing countries (Results, 2007);
3. 90% of deaths due to malaria occur in Africa, south of the Sahara, mostly among young children (RBM, 2007: *RBM means Roll Back Malaria*).

Add measles, pneumonia, and diarrhoeal disease, and you have the whole panorama of the diseases of poverty-six in all, according to the WHO. These, along with complications of childbirth, kill 14 million people a year (Results, 2007). These are individuals and societies which have neither the economic resources nor the technical expertise or manpower to handle the epidemic proportions that these, and related, diseases and disabilities assume in such vulnerable groups. This is in spite of the fact that all these six diseases of poverty can be prevented or treated for a small amount of money. For instance, medicines to treat acute malaria cost just pennies, and a measles vaccine costs just 26 cents (Results, 2007).

Poverty and disease are involved in a vicious downward spiral, each aiding and abetting the other. Poverty is an inveterate consequence and cause of ill health (Klugamn, 2002). Diseases of poverty increase poverty, and poverty, in turn, increases the chances of developing the diseases of poverty. The World Health Organization calls them “diseases of poverty” because they primarily affect the poor, and they worsen poverty's toll (Results, 2007). Often the hapless patient, and his eager but resourceless caregiver, is sucked into this vortex with no redemption in sight-and it does not take very long for an eager-resourceless caregiver to become an indolent-resourceless one. Callous regimes, scarce/overburdened medical facilities, and emotionally distanced haves in such societies only serve to seal the fate of the victims.

The interplay of these diseases of poverty is substantial and can hardly be overlooked. We know how TB compounds AIDS. TB and HIV are synergistic infections: HIV infection increases the rate of activation of latent TB infection and speeds progression of TB. TB accelerates the progression of AIDS by increasing the rate of HIV replication (NIAID Division of AIDS Research, 2007). We also know how malnutrition compounds TB. “TB is associated with poverty, overcrowding, alcoholism, stress, drug addiction and malnutrition… The disease spreads easily in overcrowded, badly ventilated places and among people who are undernourished.” (NICUS, 2007). We also cannot forget how all three, TB, HIV/AIDS and malnutrition, are dynamically interlinked with each other and with their overlord, poverty itself.

Infectious, communicable and deficiency diseases, which are aplenty in such populaces, further add to the agony. RTIs, Hansen's disease, perinatal deaths, kwashiorkor, marasmus, anaemia, vitamin deficiencies, iodine deficiency goitres, etc. are endemic in many such populations.

The social dimension of poverty can hardly be discounted. “…no social phenomenon is as comprehensive in its assault on human rights as poverty. Poverty erodes or nullifies economic and social rights such as the right to health, adequate housing, food and safe water, and the right to education” (OHCHR, 2007). Alcoholism, drug abuse, chronic mental disorders, sociopathy, beggary, violence in family and neighbourhoods, child labour, physical abuse and neglect of the female (especially the female child), commercial sex-all these, while they may impact any strata of society, leave their greatest trail of devastation among the impoverished.

## Poverty and Income/Capability/Optimism Deprivation

More importantly, the poor, assailed by life's vicissitudes and society's callousness, may learn to accept their fate and sink further into the morass of poverty, disease and deprivation. A greatly reduced self-esteem, with a feeling of being trapped in a helpless situation, with no succour in sight, adds to the crippling effect of poverty-disease-deprivation on human existence. Poverty is not just *income* deprivation but *capability* deprivation as well (Sen, 2001; p87-110). Millions of people living in the third world are still “unfree,” “denied elementary freedom and. imprisoned in one way or another by economic poverty, social deprivation, political tyranny, or cultural authoritarianism” (ibid, inner flap).

There is a distinction between lack of income and lack of capacity (Sen, 2001). Poor people acutely feel their powerlessness and insecurity, their vulnerability and lack of dignity. Rather than taking decisions for themselves, they are subject to the decisions of others in nearly all aspects of their lives. Their lack of education or technical skills holds them back. Poor health may mean that employment is erratic and low-paid. Their very poverty excludes them from the means of escaping it. Their attempts even to supply basic needs meet persistent obstacles, economic or social, obdurate or whimsical, legal or customary. Violence is an ever-present threat, especially to women.

The poorest use what resources they have, and considerable resourcefulness, in their struggle to survive. For the poor, innovation means risk, and risk can be fatal. Helping them improve their capacities calls for imagination as well as compassion (UNFPA State of the World's Population, 2007b; parenthesis added).

Equally importantly, along with *income* and *capability* deprivation, poverty also means *optimism deprivation*. Let us explain what we mean thereby. The will or motivation to fight poverty, the urge to escape its shackles, the hope that the fight will succeed one day-this *optimism* is lost due to subsistence living and the daily fight for survival. There seems to be no cause for cheer, no redemption around the corner, no way out, howsoever much the person struggles. A trapped helpless feeling, which grows on the person, aided and abetted at every step by the life situation around-this is what mainly sustains the poverty-disease-deprivation spiral. It is this *optimism deprivation* that may be a salient feature of the depression that overwhelms such individuals, adds to resource deprivation and income deprivation and, finally, does the person in. It is only those who do not suffer from optimism deprivation, in spite of suffering from the other two forms of deprivation, who manage to break free of the shackles of the poverty-disease-deprivation spiral and the concomitant depression. The examples of those who do break out of these shackles are few, but they are worthy exemplars in poverty eradication awareness programmes. The examples of those who do not escape these shackles are umpteen and they only add to the optimism deprivation in the rest.

A striking example of this phenomenon is the recent spate of farmer suicides in various parts of India. It is overtly the result of crippling poverty and loan recoveries by blood-sucking moneylenders and others. “There have been some 250 farm suicides in just the first three months of this year. Things could be a lot worse after June. And, as always, the farm suicides are a symptom of the crisis, not its cause” (Sainath, 2007). But more importantly, it is a state of feeling cornered and alienated, with no hope of escape except by escape from life itself. This is a tragic consequence of optimism deprivation-the combined result of poverty, mounting debt, untreated depression, and the callousness of regimes ensconced in their secretariats in big cities, gloating over their poverty eradication programmes, but with nothing tangible to offer when crops go bust or the prices of crops plummet. Mounting debts, fall of financial status, and loss of social reputation often result in “domino-suicides” in such populaces: “Though a sudden fall in financial status and loss of social reputation, along with family problems and illness, are among the major causes of suicides in Kerala, domino-suicides by farmers who are unable to cope with mounting debts and crop losses in such a short period constitute a new trend in the State” (Krishnakumar, 2004). The point is that many suffer from debts, fall in financial status, and loss of social reputation, but not all of them commit suicide. It is worth exploring whether the depression concomitant to the total loss of optimism is what makes those with vulnerable personalities take the extreme step.

The ones who survive often do so because of a sense of duty to survivors or because the hold of religion is strong enough to prevent such an extreme step. But what a futile survival it is, a fringe existence, often a big relief when it gets extinguished. These farmer deaths only serve to italicise the many more such poverty-related deaths that are the bane of impoverished societies-dowry deaths, female infanticide and foeticide, etc., all preventable but tragically inevitable; these are lives that prize wealth and existence, but do not have the means to connect with either.

The ones who not only survive but also emerge stronger are those who fight the conditions and the system; it is their sense of optimism about the future and about life that sustains them in this fight. It would be interesting to study optimism and its positive role in those who survive or even prosper (Seligman *et al.,* 2005) and also its absence in populations that succumb, especially so in the light of fresh evidence that MRI scans of optimistic people show activation of the rostral anterior cingulate cortex (rACC), which seems to malfunction in people suffering from depression (Sharot *et al*., 2007; see also Borenstein, 2007; Dunham, 2007).

Humans expect positive events in the future even when there is no evidence to support such expectations. For example, people expect to live longer and be healthier than average, they underestimate their likelihood of getting a divorce, and overestimate their prospects for success on the job market. We examined how the brain generates this pervasive optimism bias. Here we report that this tendency was related specifically to enhanced activation in the amygdala and in the rostral anterior cingulate cortex when imagining positive future events relative to negative ones, suggesting a key role for areas involved in monitoring emotional salience in mediating the optimism bias. These are the same regions that show irregularities in depression, which has been related to pessimism. Across individuals, activity in the rostral anterior cingulate cortex was correlated with trait optimism. The current study highlights how the brain may generate the tendency to engage in the projection of positive future events, suggesting that the effective integration and regulation of emotional and autobiographical information supports the projection of positive future events in healthy individuals, and is related to optimism. (Sharot et al., 2007).

The effect of chronic poverty on the rACC, of capability deprivation, optimism deprivation, and of related depression is worth serious study to determine how each impacts the other. Standardised scales that measure optimism deprivation are worth constructing, as are studies to determine its importance as an independent variable. Potential suicide markers may be identified by such means in vulnerable populaces. Trait optimism, the effect on rACC of retained optimism in spite of crippling poverty and concomitant depression, is worth study too.

## Millennium Development Goals

The United Nations Secretary-General in 2002 commissioned the Millennium Project to recommend a concrete action plan for the world to reverse the grinding poverty, hunger and disease affecting billions of people. It presented its final report, *Investing in Development: A Practical Plan to Achieve the Millennium Development Goals*, to the Secretary-General in January 2005 (Millennium Project, 2002-2006).

We have the opportunity in the coming decade to cut world poverty by half. Billions more people could enjoy the fruits of the global economy. Tens of millions of lives can be saved. The practical solutions exist. The political framework is established. And for the first time, the cost is utterly affordable. Whatever one's motivation for attacking the crisis of extreme poverty-human rights, religious values, security, fiscal prudence, ideology-the solutions are the same. All that is needed is action (Millennium Project, 2002-2006).

The Millennium Project has eight extremely honourable and worthwhile goals:

- Eradicate extreme poverty and hunger
- Achieve universal primary education
- Promote gender equality and empower women
- Reduce child mortality
- Improve maternal health
- Combat HIV/AIDS, malaria, and other diseases
- Ensure environmental sustainability
- Develop a global partnership for development (UN Millennium Development Goals, 2007).

One of the greatest challenges for the world today is to actualise these eight goals. While the first six mainly concern developing societies, the final two concern all populations everywhere. Even the first six are of concern to those amongst the haves whose social consciousness has not been extinguished.

As one immediately realises, these goals are mainly targeted at developing societies. What this paper intends to show is that the developed world also has a goal to achieve:

- Control of lifestyle diseases and the promotion of longevity *with* well-being.

This could become a worthy ninth addition to the eight goals mentioned above. But before we agree, let us first understand the problems of the haves.

## No Great Cause to Cheer in the Haves

Escaping the clutches of diseases of poverty does not necessarily mean any great cause for cheer in the haves. They have other problems on hand. Lifestyle diseases take their own toll. These are diseases “associated with the way a person or group of people lives. Lifestyle diseases include atherosclerosis, heart disease, and stroke; obesity and type 2 diabetes; and diseases associated with smoking and alcohol and drug abuse” (MedicineNet, 2007).

What is intriguing about lifestyle diseases is that they appear to become ever more widespread as countries become more industrialized (Natural Perspective on Health, 2007). They are the result of an inappropriate relationship of people with their environment (ibid). More importantly, they are potentially preventable and can be lowered by changes in diet, lifestyle and environment (ibid). For example, regular physical activity helps prevent obesity, heart disease, hypertension, diabetes, colon cancer, and premature mortality (MedicineNet, 2007). Equally important, lifestyle diseases are insidious, may take years to develop and, once encountered, do not lend themselves easily to cure (Natural Perspective on Health, 2007).

Diseases like hypertension, diabetes, depression, and ischaemic heart disease can be quitet pernicious, especially when uncontrolled and accompanied by lifestyle stresses, and those afflicted often have to wage a life-long battle to redeem whatever sense of well-being they can in the wake of such chronic afflictions. While life expectancy may have increased in the haves, and infant and maternal mortality reduced, it has not necessarily ensured that well-being results. Divorces are rampant (MedlinePlus, 2007), with pre-divorce interpersonal and legal stresses, post-divorce life adjustments, single mothers/parents and their problems, child psychopathology due to pressured parenting responsibilities on such single parents/mothers etc. (Compas and Williams, 1990; Dumka, Roosa and Jackson, 1997; Dunn, O'Connor and Cheng, 2005). Also causing great concern are health-compromising behaviours like addictive smoking (Pure Insight, 2002; Mansvelder and McGehee, 2000; Mansvelder, Keath and McGehee, 2002; Fiore *et al*., 2004; Barr and Curry, 2004; Turner *et al*., 2004; Sussman, Dent and Stacy, 2002; Curry *et al*., 2006); problem drinking (Enoch and Goldman, 2002; AAFP, 2004); Internet addiction (Virtual-Addiction.com, 2007; DeAngelis, 2000), intensive mobile phone use, especially amongst adolescents (Koivusilta, Lintonen and Rimpela, 2005); use of anabolic steroids and sexual offences in adolescents (Holmberg and Berg-Kelly, 2002); overeating and consequent obesity (Blissmer *et al*., 2006; and especially in diabetes, Vallis *et al*., 2003); and unprotected sexual promiscuity, with resultant STDs and HIV/AIDS (and how community involvement can help, Jesus, 2002). There are also the debilitating conditions like arthritis (Haaz, 2005), cancers (American Cancer Society, 2005), strokes (American Stroke Association, 2003), myocardial infarctions (University of Maryland Medical Center [UMMC], 2007), uncontrolled diabetes (National Diabetes Information Clearinghouse [NDIC], 2007), hypertension (Oparil and Calhoun, 1998; Brady, Spiro III and Gaziano, 2005), major depression (NIMH, 2007), dementias (NINDS, 2007) etc. While the means to enjoy life have multiplied, so have vehicular accidents (Reinhardt, 2004), drunken driving (WHO, 2007a; p71), and related disabilities (WHO, 2007b; p49). The global burden due to vehicular accidents is projected to increase phenomenally (Murray and Lopez, 1996a, 1996b); it is already the foremost cause of death in 15-19 year olds (Singh and Singh, 2004). This is the reason the WHO organised the World Health Day 2004 on 7 April of that year and dedicated it to the theme of road safety with the slogan, “Road Safety is No Accident.”

The stresses and consequences of an expensive lifestyle, without the means to maintain it, and the pressured work-style to maintain it (Scott, 2007; Miller, 2004); the problems of work-life imbalance and how to reduce it (Mayo Clinic Staff, 2006); workaholics and the desire to keep working compulsively (Workaholics Anonymous, 2007); the excessive preoccupation with making money, achieving fame and influence quickly and without ethical qualms-ethical considerations being dropped faster than clothes in an X-rated movie-and a mind-set preoccupied with activities aimed at achieving mainly such goals in life, including the “casting couch,” multiple sexual liaisons, and networking with the ethically compromised; multiple loan repayments, multiple credit card usage and debt (Drentea and Lavrakas, 2000), loan recovery proceedings and related legal hassles; debt and health (Dossey, 2007); the susceptibility to illness and mental health problems due to life events (Holmes and Rahe, 1967; Horowitz *et al*., 1977)-all these have still not engaged the attention of serious research with the intensity they should have considering their implications for disease causation/exacerbation/perpetuation. However, the effects are there for all to see, if only they are perceptive enough. It is time rigorous studies that address these issues engaged the attention of serious researchers.

The case of the have-nots of such societies (and this includes the chronically mentally ill)-with their own problems of homelessness, lack of education, joblessness, drug abuse, delinquency, and crime involvement hardly add to the glory of human existence. Their activities bring no cheer, either to themselves or to the affluent around them, who often have to bear the brunt of the have-nots’ ire at their affluence.

## Societies in Transition and Societies in Distress

We see, therefore, that those in developing societies have to fight their battles mainly against infectious and communicable diseases, while those in the developed societies battle mainly against the lifestyle ones. The fight against infectious diseases and poverty is the first battle to be won for the developing world, but lifestyle diseases are waiting in the wings. As many in the developed society seldom realise, here as elsewhere, lifestyle diseases often creep up on the populace surreptitiously. Life's opportunities to achieve and enjoy may increase with jet-setting and ladder-climbing in high-paying professions. But in creeps diabetes, hypertension, heart attack, or a cerebrovascular stroke to apply the brakes on further ambitions-often at a time when the situation is best suited for fulfilling them. The stress of juggling career shifts, of obsessive money-multiplying investment schemes, and of addicting casual emotional entanglements and sexual liaisons-all add to a roller-coaster existence, which is the bane of affluence. Negative emotions and behaviours like dominance, hostility, resentment, cynicism, antagonistic behaviour, nervousness, pessimism, etc. take their toll on health and well-being. Professional burnout is often a concomitant problem.*

The rootedness in a value system is an inconvenience forsaken pretty early. Prayer and philanthropy are poor means to assuage the tempestuous inner guilt that results from the lack of such internal rootedness. It is here that value education early on, and professional ethics all through life, assume importance as guidelines for achieving a success that is not only experienced but also enjoyed. It's only a solid foundation in values that allows for such enjoyment:

While success is important, it can become enduring only if it is based on a strong foundation of values. Define what you stand for as early as possible, and do not compromise with it for any reason. You can't enjoy the fruits of success if you have to argue with your own conscience (Premji, 2004).

In the absence of such rootedness, professional burnout, psychosomatic ailments, and stress thrive at the cost of well-being. Some attention to these entities would be worthwhile here.

## Professional Burnout, Psychosomatic Medicine and Stress

### 1. Professional burnout

Professional burnout, first introduced as a concept by Freudenberger (1974), has been defined as, “an experience of physical, emotional, and mental exhaustion caused by long-term involvement in situations that are emotionally demanding” (Kuremyr *et al*., 1994). Another useful way of looking at it is as a syndrome of emotional exhaustion (with tiredness, somatic symptoms, decreased emotional resources, and a feeling that one has nothing left to give to others); depersonalization (developing negative, cynical attitudes and impersonal feelings towards clients, treating people as objects); and lack of feelings of personal accomplishment (feelings of incompetence, inefficiency, and inadequacy) (Lee and Ashforth, 1990).

Professional burnout has been well studied (Maslach, Schaufeli and Leiter, 2001; Shirom, 2003; Shirom *et al*., 2005; Cordes and Dougherty, 1993; Schaufeli and Buunk, 2003; Schaufeli and Enzmann, 1998; Kahill, 1988). It has an impact on mental health (Toker *et al*., 2005; Cordes and Dougherty, 1993; Maslach, Schaufeli and Leiter, 2001; Schaufeli and Enzmann, 1998; Shirom and Ezrachi, 2003; Toker *et al*., 2005). The following are promising areas of current research into the varied connections between mental health, work/lifestyle stress and their psychophysiological manifestations: the relation between cytokines and depression (Corcos *et al*., 2002); between depression and elevated C-reactive protein (Danner *et al*., 2003; Douglas *et al*., 2004; Ford and Erlinger, 2004); between chronic diseases and fatigue in the working population (Franssen *et al*., 2003); the role of a depression-management office system in community practice (Dietrich, 2003); physiological correlates of burnout among women (Grossi *et al*., 2003) and those with CHD (Hallman *et al*., 2003); stress, burnout, and coping differences between women with and without CHD (Hallman *et al*., 2003); relationship between job stress and fibrinogen (Theorell, 2002); vital exhaustion in industrial workers and reduced glucocorticoid sensitivity of monocyte interleukin-6 production (Wirtz *et al*., 2003), and between depressive and vital exhaustion symptomatology post-myocardial infarction (Wojciechowski *et al*., 2000); work stress as an important determinant of CHD among working-age populations, mediated through indirect effects on health behaviours and direct effects on neuroendocrine stress pathways (Chandola *et al*., 2008).

Burnout also has effects on physical health, including on diseases like type 2 diabetes (Melamed, Shirom and Froom, 2003) and cardiovascular disease (Appels, 1988; Appels and Schouten, 1991a; Hallman *et al*., 2003; Melamed, Kushnir and Shirom, 1992). Waking up exhausted has been suggested to be a risk indicator of myocardial infarction (Appels and Schouten, 1991b). Burnout causes impaired fertility (Sheiner *et al*., 2002); promotes adverse health behaviours, including smoking, lack of exercise, and excessive calorie intake (Gorter, Eijkman and Hoogstraten, 2000; Melamed, Kushnir and Shirom, 1992; Schaufeli and Enzmann, 1998); and results in poor self-rated health (Gorter, Eijkman and Hoogstraten, 2000; Halford, Anderzen and Arnetz, 2003; Kahill, 1988).

Burnout in medical and paramedical professionals is also being studied. Physician well-being (West and Shanafelt, 2007; Dunn *et al*., 2007; Williams, 2007a) and burnout (South, 2006; Halbesleben and Rathert, 2008) is receiving well-deserved research attention, including in intensive care staff (Cubrilo-Turek, Urek, and Urek, 2006), in dialysis centres (Argentero, Dell'Olivo and Ferretti, 2008), among anaesthesiologists (Shchelkova and Mazurok., 2007), women in medicine (Williams, 2007b), emergency physicians (Shahid, 2007), surgical oncologists (Balch and Copeland, 2007; Kuerer *et al*., 2007), resident doctors (Thomas, 2004), psychiatrists (Fothergill, Edwards and Burnard, 2004; Kumar, 2007), acquired immune deficiency syndrome (AIDS) care nursing staff (Hayter, 1999), community mental health nurses (Edwards *et al*., 2006) etc.

Measures and intervention programmes for burnout are being studied and developed too. To ensure objectivity, two measures have been constructed: 1. The Maslach Burnout Inventory-General Survey (MBI-GS), the more popular measure of burnout (Maslach and Jackson, 1986); and 2. the more recent Shirom-Melamed Burnout Measure (SMBM) (Shirom and Melamed, 2006). Other nuances of burnout being explored are: comparisons between two rehabilitation interventions (Hätinen *et al*., 2007) and examination of burnout in psychosocial rehabilitation teams and its effects on patient satisfaction (Garman, Corrigan and Morris, 2002) etc.

### 2. Psychosomatic medicine and stress

The major thrust of psychosomatic medicine in the last decade has been towards unraveling the varied manifestations of stress, including in cardiovascular disease (Black and Garbutt, 2002); the role of vital exhaustion as a precursor of myocardial infarction (Appels, 1988); depression and coronary disease (Appels *et al*., 2000; von Kanel *et al*., 2001); fibrinogen and women's health (Vorster, 1999); psychosocial factors in the development of coronary artery disease (Strike and Steptoe, 2004); clinical depression and inflammatory risk markers for coronary heart disease (Miller *et al*., 2002); inflammatory markers and depressed mood in older persons (Penninx *et al*., 2003); cytokine-associated emotional and cognitive disturbances in humans (Reichenberg *et al*., 2001); impact of psychological factors on the pathogenesis of cardiovascular disease and implications for therapy (Rozanski., Blumenthal and Kaplan, 1999); depression as a predictor for coronary heart disease (Rugulies, 2002); the role of emotion on pathways to positive health (Ryff and Singer, 2003); depressive symptoms and metabolic risk (McCaffery *et al*., 2003); pathways linking depression, adiposity, and inflammatory markers in healthy young adults (Miller *et al*., 2003); how depression is bad for the failing heart (Joynt, Whellan and O'Connor, 2004); psychoneuroimmunology in emotions, morbidity, and mortality (Kiecolt-Glaser *et al*., 2002); depression and the metabolic syndrome in young adults (Kinder *et al.,* 2004); emotion and immunity (Koh, 1998); the integration of cardiovascular behavioural medicine and psychoneuroimmunology (Kop, 2003); inflammation and coagulation factors in persons >65 years of age with symptoms of depression but without evidence of myocardial ischemia (Kop *et al.*, 2002); effects of psychological and social factors on coronary heart disease (Krantz and McCeney, 2002; Kuper, Marmot and Hemingway, 2002; Hallman *et al*., 2001); negative emotions and coronary heart disease (Kubzansky and Kawachi, 2000; Niaura *et al*., 2002); the relationship between antagonistic behaviour, dominance, attitudinal hostility, and coronary heart disease (CHD) (Siegman *et al*., 2000); anger and hostility as predictors for the development of atrial fibrillation in men (Eaker *et al*., 2004); emerging risk factors for atherosclerotic vascular disease (Hackam and Anand, 2003); influence of depressive mood on the association of CRP and obesity (Ladwig *et al*., 2003); depression, anxiety, and associated health status (Lubetkin, Jia and Gold, 2003); inflammatory markers and risk of developing type 2 diabetes in women (Hu *et al*., 2004) etc.

The major challenge before psychosomatic research in the next decade will be to tie up the loose ends, identify definite aetiologic factors, and lay down clear-cut therapeutic guidelines to prevent and treat lifestyle diseases. This will be of the greatest importance in our search for a longevity that also ensures well-being.

### 3. Stress, lifestyle, and well-being

There are ever-multiplying numbers of individuals whose well-being is compromised due to the lifestyle diseases mentioned above-the result of faulty lifestyles and the consequent crippling stress. But it serves no one's purpose to understand them as such. And hence they go unattended, even as the conditions that result therefrom are intensively treated. What a parody. So much treatment, such fancy infrastructure, so much research-but no realisation of the obvious. Simply because it does not serve the purpose of the establishment to accept it as such.

There are two main approaches to lifestyle diseases (and a third much needed addition):

1. *Medical approach:* The first we may call the medical approach. Here, the major effort of mainstream medicine is to get tests and procedures done, get people periodically examined, check their serum cholesterol/creatinine/blood pressure/blood glucose etc., get them to understand what is good and bad cholesterol, get the cardiac stress test done etc. They may prescribe cholesterol/triglycerides/blood-pressure/blood sugar-lowering drugs, as also drugs for angina and prevention of MI. This is the most vigorously followed approach, and receives the greatest clinical and research attention for obvious reasons-it adheres closely to the predominant medical model of diagnosis, pathology, and treatment.
  Different from, but connected to, the above are the two lifestyle approaches we will try and outline below-the actionable lifestyle approach and the attitudinal change lifestyle approach.
2. *Actionable lifestyle approach*: This approach is given token emphasis by most and serious attention by a few. Here, the doctor may venture to concentrate on advice on actionable methods to reduce lifestyle diseases-for example, reduce weight, cut down on smoking, moderate alcohol intake, start exercise, go for a walk etc. See for example:

I. A standard recommendation (Iestra *et al*., 2005: [Table 1]) to:

1. Stop smoking;
2. Engage in moderate intensive physical activity (for 30 min on at least 5, but preferably all, days of the week);
3. If you use alcohol: do so in moderation (maximum 2 alcoholic drinks per day for women and maximum 3 drinks per day for men);
4. Maintain or attain a healthy body weight (BMI < 25 kg/m2); obese patients (BMI >30 kg/m2) should try to lose 10-15% of their current body weight;
5. Limit your saturated fat intake (to a maximum of 10 energy%) and the intake of trans fatty acids (to a maximum of 1 energy%);
6. Consume fish regularly (at least 1 and preferably 2 portions of oily fish per week);
7. Consume sufficient amounts of fruits and vegetables (400 g/d);
8. Use sufficient fibre-containing grain products, legumes, and/or nuts (3 U/d); and
9. Reduce your salt intake (to maximal 2400 mg/d).

II.Or, see the Mayo Clinic 5-strategies recommendation to prevent heart disease:

1. Don't smoke or use tobacco products;
2. Get active;
3. Eat a heart healthy diet;
4. Maintain a healthy weight;
5. Get regular health screenings (MayoClinic.com, 2007).

III. In two recent studies, for example, increased fitness was associated with 50-70% reductions in all-cause mortality (Kokkinos *et al*., 2008), and staying active and drinking moderately was the key to a long life (Pederson *et al*., 2008).

All such recommendations are fine and practical ways to stay healthy. But often it is realised that such suggestions are not accepted/implemented by those who should be doing so, because they are attempted in isolation of attitudinal changes in one's lifestyle. Let us see what that means.

3. *The attitudinal change lifestyle approach:* This third approach is the one that needs to be understood and implemented to bring lifestyle diseases under control, reduce the need for approaching a medical facility/practitioner, and accentuate the benefits from, and adherence to, the lifestyle approach. Here we concentrate on the traits and attitudes that predominate in individuals who have lifestyle diseases. If one continues to remain hostile, unduly dominant, constantly angry, forever nursing negative emotions, one can hardly follow or benefit from simple dietary and exercise schedules. Either they are abandoned or, worse, serve only to give a false sense of protection in the absence of attitudinal changes. Similarly, unless such attitudinal changes are brought about, the medical approach will have to be used indefinitely and its use escalated. That may serve the interests of the medical establishment but it hardly serves the interests of the patient.

To study and incorporate personality variables and psychosocial risk factors into disease prevention and treatment and focus intense research attention on these variables does not seem to be high on the list of priorities of the opinion makers today, although honest soul-searching cannot keep them immune to this realisation. Further, to say: change your lifestyle, apply the brakes, step down from the “work-treadmill,” simplify your life, downsize career ambitions, avoid over-consumption, control your anger and your avarice, spend time with your family, be compassionate to others, tune in to your inner self-all this appears fine, but it is hardly what a doctor can prescribe, used as he is to the convenience of his prescription pad and the friendly pharmaceutical companies that offer him newer opportunities to use it. So he leaves the dispensing of this advice to preachers and God-men, who thereby develop their own burgeoning “practice.” And then the men of medicine crib when patients run for succour to such God-men and other esoteric healers. Moreover, such advice has hardly been scientifically validated by rigorous studies (as opposed to the benefits of most so-called medical procedures, as also of diet and exercise, which have been validated over and over again); and that's always a convenient handle. How could they be validated, if they do not enter the consciousness of the prescribing physician or the researcher? Unless, of course, he himself suffers from a lifestyle disease; then he is likely to adopt all these for himself. He will not only take drugs and undergo procedures, not only diet and exercise, but also slow down, simplify his life, try to tune in to his inner self, control his anger and his greed, be compassionate to others, etc. He will start Yoga, meditation, Vipassana, attempt therapies from alternative and complementary medicine, etc. Depending on his level of realisation, and the ability to convert it into actionable advice for his clientele, he may or may not advise them to act similarly. For, the clinic has to run as well. The first cluster of advice, based on the prescription pad, ensures it; the second and third, based on diet/exercise and attitudinal changes, respectively, do not necessarily contribute.

So, the prescription pad continues to prevail over lifestyle-change counselling or research. And over the realisation that it is the ubiquitous lifestyle stress that causes a pervasive sympathetic overload; an overload that results in such stress hormone outpourings as wrecks the otherwise robust internal organ systems and their homeostasis.

It is time this was realised and as much serious research attention given to the third approach as is given to the first and the second.

## The Way Out

It is thus obvious that morbidity, disability, and death assail all three, the ones with infectious diseases, the ones with diseases of poverty, and the ones with lifestyle diseases. If it is bacteria in their various forms that are the culprit in infectious diseases, it is poverty/deprivation in its various manifestations that is the culprit in poverty-related diseases; and it is stress in its various avatars that is the culprit in lifestyle diseases. It is as though poverty and lifestyle stress have become the modern “bacteria” of the developing and developed societies respectively.

There is a way out of this morass. For all parties.

### 1. For the Have-Nots

For those societies afflicted with diseases of poverty, of course, the prime concern is to wriggle out of the deadly grip of poverty-disease-helplessness. But, while so doing, be careful not to land in the lap of lifestyle diseases. This they can do only if, individually and collectively (but especially individually), they realise the enormous potential of the human spirit and are ready to trudge the long way uphill against callousness, deprivation and exploitation. They must do so without nursing anger, resentment, and hostility towards the haves. Simply because these emotions will never allow them inner peace; they will only make them more amenable to the lifestyle diseases waiting to take over.

### 2. For the Haves

For the haves, the need is to seek inner rootedness, to seek the healthy pursuit of self-esteem (DuBois and Flay, 2004), to ask science not only to give them new pills for new ills but to define and study what is inner peace, what is the connection between spirituality and health, what is well-being, how negative emotions hamper health and how positive ones promote it, what is self-actualisation, what prevents disease, what leads to longevity, what attitudes help cope with chronic sicknesses, how can sicknesses be reversed (and not just treated). To confront the man of science who keeps accusing the man of religion of talking without evidence, and instead forcing the former to collaborate with the latter to find ways of mitigating human distress by finding such evidence. To force the man of medicine to stop medicalising everything or, if he must, to offer both preventive and curative (not just controlling/ameliorating) therapies. To find how alternative/complementary medicines work and, if they actually do, to adopt such procedures for his patient population. To avoid restricting himself to the treatment of just the disease but, rather, to concentrate on its prevention; to help a person function at his optimum in spite of his disease. For that, the physician will first have to realise that it is a person who has a disease, and it is not just a case of a disease afflicting a person. The cognitive shift that this entails is the only way the practitioner of biomedicine can become a catalyst not only in removing/controlling the diseases of poverty and lifestyle, but also become a catalyst in our search for that elusive goal-human development and well-being. For this, it is man, and his development and welfare, which should be the measure of all things. Scientific progress, economic development and social order are meant to promote human development, not the other way around.

It is heartening to note that in the last few decades some scientific attention, though belated and grudgingly given, is getting focussed on the concept of well-being, the means to achieve longevity, and the need to make life simple. Some attention to these areas may be pertinent here.

## Well-being

The goal of all health-related measures is not just treatment of disease, but also its effective control, eventual cure, and prevention. More important, it is the promotion of well-being, an amorphous concept getting more standardized with scales like the Positive and Negative Affect Scale (PANAS) (Watson and Clark, 1997), which measures the presence of positive and absence of negative emotions; the Temperament and Character Inventory (Cloninger, Svrakic and Przybeck, 1993), which measures mature character traits, including self-directedness, cooperativeness and self-transcendence; the Satisfaction With Life Scale (SWLS) (Pavot and Diener, 1993), which measures life satisfaction or quality of life, and character strengths and virtues, such as hope, compassion, and courage (Peterson and Seligman, 2004).

What characterises well-being? It is mainly positive emotions, character strengths, virtues, and life satisfaction, which is the result of growth in self-awareness (Cloninger, 2004). It is also the result of letting go of all struggles, working in the service of others, and growing in awareness. Self-transcendence-man's search for what is beyond individual human existence-is also crucial for the development of resilience and maintenance of well-being (Cloninger, 2004).

Self-transcendence has been shown to have a strong neurobiological basis in human evolution, with unique genetic determinants (Gillespie *et al*., 2003) and serotonergic neurotransmission (Borg *et al*., 2003). Self-transcendence is also important for the preservation of grey matter for meta-cognition as people age (Kaasinen, 2005). Augmentation of cognitive-behavioural therapy with increased awareness reduces relapses and recurrence rates in therapy for a wide range of disorders compared to cognitive-behavioural therapy alone (Cloninger, 2006; D'Souza and Rodrigo, 2004; Fava *et al*., 2005; Teasdale *et al*., 2002).

Positive psychology has been preoccupied with the cognitive and social aspects of well-being, neglecting both its neurobiological and its spiritual roots (Cloninger, 2005). The foundation for personal well-being is the self-awareness that each being is an inseparable part of a universal unity of being (Cloninger, 2008). Thinking beyond individual human existence, understanding and establishing transpersonal connections are essential to well-being. Here thoughts of the essential unity of all existence, like for example the thoughts of Sri Aurobindo, assume importance (Singh and Singh, 2009; forthcoming). This does not necessarily need religion, although religion facilitates such a realisation. The role of positive emotions and spirituality that psychiatry has neglected, and religion itself as “the portal through which positive emotions are brought into conscious attention” (Vaillant, 2008) deserves the close attention of serious science researchers. Even agnostics and atheists have a spiritual perspective that helps them understand the purpose and meaning of their life (Hay, 2007).

In our quest for well-being, the connection between competition and territoriality on the one hand, and poverty, conflicts and war on the other also deserve our careful attention:

What do deep primitive beliefs about the primacy of competition and territoriality have to do with poverty, conflicts and wars? All are rooted in ancient human fears-of scarcity, of attacks by wild animals or other fearful bands of humans. Rooting out these fears - deeply coded in our “us-versus-them” political and economic textbooks- is the essential task of our generation. We must move beyond this economics of our early reptilian brains- to include the economics of our hearts and forebrains! These old fears underlie today's continuing cycles of oppression, poverty, violence, revenge and terrorism. Indeed, if we humans do not root out these now-dysfunctional old fears, we will destroy each other. Politicians frequently use fear to manipulate consent. Yet fear can be counterproductive. Franklin D. Roosevelt during the Great Depression in the US proclaimed that we have nothing to fear but fear itself! (Henderson, 2005).

How we move from competition and confrontation to cooperation and consensus, without blunting the entrepreneurial edge, is the great challenge before mankind today.

Closely connected with the concept of well-being is the search for longevity, which we may now briefly survey.

## Longevity

Based on the life of centenarians, Maurice Ernest (Ernest, 2006), identified the following key factors to long life:

1. Eat frugally
2. Exercise and get plenty of fresh air
3. Choose a congenial occupation
4. Develop a placid and easy-going personality
5. Maintain a high level of personal hygiene
6. Drink wholesome liquids
7. Abstain from stimulants and sedatives
8. Get plenty of rest
9. Have a bowel movement once a day
10. Live in a temperate climate
11. Enjoy a reasonable sex life
12. Get proper medical attention in case of illness

Studies of the lives of those who live long, for example in Abkhasia (Chopra, 1993a; p231-260), as well as of other centenarians, show that they are autonomous, adaptable, have lack of high ambition, live quiet and independent lives, are happy with their job, family, religion; have few regrets, have high appreciation for the simple experiences/pleasures of life; and have a sense of self-sufficiency (Chopra, 1993b; p196-216)

What stands out from such observations/studies are the following:

1. *Moderation of lifestyle:* eating frugally; choosing a congenial occupation; developing a placid and easy going personality; abstaining from stimulants and sedatives; living in a temperate climate; enjoying a reasonable sex life; lack of high ambition; living quiet and independent lives; being happy with their job, family, religion; having few regrets; having high appreciation for simple experiences/pleasures of life;
2. *Care for physical self:* eating frugally, and therefore not becoming overweight; exercising and getting plenty of fresh air; maintaining a high level of personal hygiene; drinking wholesome liquids; having a bowel movement once a day; getting proper medical attention in case of illness;
3. *Self-sufficiency and simplicity:* living quiet and independent lives; lack of high ambition; having high appreciation for the simple experiences/pleasures of life; being autonomous; having a sense of self-sufficiency.

The pattern that emerges is of an individual who cares for his physical health, does not push himself too far in competition and strife, is simple in tastes and easy-going by nature, and self-sufficient enough to survive the losses that are inevitable over one's life span: of loved ones, economic hardships, loss of power, prestige, etc. Simplicity is often the cornerstone of a life lived long, and it would be worth our while to concentrate on it here.

## The Simplicity Movement

It is heartening to note that in certain quarters vigorous steps are on to simplify life and increase awareness of the ill effects of:

1. Over-consumption or “consumption addiction”-- the “addiction to too much. and the distress of excess”;
2. Of remaining over-connected so that, “people can access us any time of the day or night, (and) we feel perpetually connected to our work”;
3. The problem of technology overload, “Being flooded with stimuli (which) is a tremendous source of stress”; and
4. “Overexposure to negative or toxic people” (WebMD, 2008. Parenthesis added.)
  and hence understand the value of
5. Reducing debt, de-cluttering one's home and downsizing one's career ambitions (WebMD, 2008).

What is this simple living? “It means many things to many people, but we are broadly defining it in lay terms as ‘The Satisfaction of Enough’” (Simple Living America, 2008). It is also defined as:

*Simple Living is a self-endorsed pattern of activities, possessions and values that is substantially free of detractions from fulfilment and sufficiency, fostered by conducive social policies* (Simple Living America, 2008)

Simple living is not about living in poverty or self-inflicted deprivation. Rather, it is about living an examined life-one in which you have determined what is important, or “enough,” for you, discarding the rest (Simple Living Network, The, 2008).

The case is strong for research that studies the findings of the “simplicity movement,” or “voluntary simplicity” as it is also called; the work that spawned interest in simplicity, that of Duane Elgin (Elgin, 1981/1998) as well as other works (St. James, 1994; Holst, 2007); as also initiatives like Simple Living America, which is part of the CRESP (Center for Religion, Ethics & Social Policy) Centre for Transformative Action at Cornell University (http://www.getsatisfied.org/main/content/view/19/43/). In 2004, this centre co-sponsored a conference with UCLA's Neuropsychiatric Institute on “Mental Health and Simple Living: Countering the Compulsion to Consume.” The various ramifications of how simplicity impacts health needs to be further studied by other scholars at workshops and seminars across the globe.

Those who wish to live long, and well, have much to learn about lifestyle and personality modifications from writings and studies such as these.

## The Task Ahead for Each One of Us

In the light of what we have discussed so far, it is possible to visualise the task ahead for each one of us.

***Physician:*** If you are a physician, you now know the task cut out for you. You know how poverty/deprivation in the have-nots and lifestyle stresses in the haves contribute to disease causation and perpetuation in your patient population. Ask for therapies that ensure well-being and adopt measures that ensure longevity in your patient population. Look out for professional burnout and negative emotions as they impact health and cause/impact disease. Be careful before you prescribe the costly new medicine to the poor, who may drain their meagre savings to somehow comply with your prescription. Be careful to look into the anger-greed-hostility-over-consumption-immorality angle that often complicates recovery from lifestyle diseases. Advise your patients to simplify their lives. Also, stop being impersonal in your treatment, without seeming to interfere needlessly in personal matters. Be more humane, look to help the person behind the disease, even as you do not neglect being objective, for both these ensure comprehensive treatment of the disease. Know the boundaries not to be transgressed. Tell the science researchers and policy planners to clean up their acts and offer you clear-cut evidence of what works where; how to control/prevent diseases of poverty and lifestyle; how to prevent burnout; how to promote well-being; and how to ensure longevity. Refuse to be taken for a ride by marketing techniques masquerading as scientific evidence.

***Patient:*** If you are a patient, you now know the task cut out for you. Demand for definitive research to combat the diseases of poverty and those of lifestyle. Demand for research directed at the cure of diseases, not control of symptoms. Demand longevity with well-being, not just longevity alone. Simplify your life, declutter your home, downsize your ambitions, avoid excessive debts, over-consumption, and over-connectedness, and avoid over-exposure to negativity. Ask for physicians and researchers to clean up their acts. Stop being satisfied with what an archaic physician dishes out to you. Ask for, and expect, uncompromising scientific evidence for the treatment offered. Remember, treatment administered should cure sometimes, comfort always, hurt the least, harm never (Singh and Singh, 2006). Continue to have faith in your physician, expect him to do his best, expect him to be firm with you at times; but know your rights as well, so you do not get taken for a ride. Expect informed consent to be exactly that, informed, and never get coerced into unsubstantiated research or treatment. Demand, and get, your right to be handled with care and compassion, and expect your physician to give of his time and expertise liberally, even as you do not take advantage of his gentleness. Give due credit to medicine (and due cash to the medical person who makes it possible for you to enjoy its benefits). The physician is no God, true, but often treating him like one works in your favour.

***Caregiver:*** If you are a caregiver relative, you now know the task cut out for you. Expect the best from your physician, but equally important insist on landmark research to prevent/cure the different diseases. Caregiving can be exhausting, can be extremely stressful, and result in burnout. Know and learn better ways of coping with caregiving, especially in chronic debilitating conditions. The patient is suffering, and so are you. It hardly helps if you increase his suffering by nursing negative emotions, by passing caustic remarks, or by neglecting his genuine needs, for it only exacerbates his problem and, more importantly, compounds your own as a caregiver. Expect your physician to be competent, but caring too. Do not tolerate his lack of courtesy or any brusqueness. Do not accept that ethical standards are low everywhere, so how can medicine be spared. Demand high ethical standards, even as you demand high professional standards, and be ready to pay for what you get.

***Biomedical researcher:*** If you are a researcher, you now know the task cut out for you. The patients, caregivers, and physicians have had enough of your dillydallying. Give them therapies that work. Tell them the reason why they fall sick so they can prevent it. Give them clear-cut preventive measures. How does poverty interact with disease, how does lifestyle and stress affect body and mind? What roles can religion, spirituality, and alternative and complementary therapies play? Do not sneer at them for their lack of robust evidence-subject them to searching critical scrutiny, but redefine and refine your parameters to understand them too. Work towards giving therapies that cure, not just control. Study well-being, simplicity, and longevity systematically and comprehensively. Orient your researches to answer such questions. You can continue your intellectual arguments, your work for so-called scientific progress, and take care of your own career advancement with smart write-ups and speeches. But you cannot afford to neglect the hapless patient or the harassed caregiver and, thereby, the well-being of society in general, whose welfare alone is your raison d'etre.

***Writer/journalist:*** If you are a writer/journalist, you now know the task cut out for you. Ask for, and get, clear-cut evidence for therapies that work. Diseases of poverty and lifestyle grip major populations groups all over the world. What is the purpose of massive research funding and elaborate seminars, conferences, and workshops, if we are to be satisfied with only minor improvements, and there is no great movement to ensure well-being and positive health? Where even health defined as absence of disease is a very distant goal? Be on the look out for studies on well-being, simplicity and longevity, and help create a lobby for such type of research to flourish. Expose research misconduct ruthlessly, reputations be damned; but see to it that you do not become a pawn manipulated by some nefarious individuals and groups, and do not try to fish in troubled waters yourself.

***Science administrator:*** If you are a science administrator, you now know the task cut out for you. Science is meant to bring about human welfare, even as it perpetuates its own progress. There can be no respite from supporting rigorous research into the diseases of poverty in the have-nots, and diseases of lifestyle in the haves. This agenda must be firmly set, and no waylaying of this agenda should be pardoned in researchers who do flimsy work with research grants. Stop playing God, or having blue-eyed boys. Only the most urgent research and the most competent researchers should get the research grant. Be especially aware of neglected diseases and research areas that may not be fashionable to work in, but are the real need of the people. Systematic and comprehensive study of well-being, burnout, simplicity and longevity must be promoted. Lobby with science administrators worldwide to pressure governments and bureaucrats to make health planning and care a priority on their agenda, along with poverty eradication programmes, and the means to make lifestyle changes possible.

***Policy maker:*** If you are in a position to plan policies or execute them in government, you now know the task cut out for you. Stop finding reasons to put health and education on the back burner. Just think of your own self: if diseases of poverty won't get you, diseases of lifestyle definitely will. Systematic and comprehensive study of well-being, burnout, simplicity and longevity is the need of the hour. It is even in your own self-interest that unconflicted research finds preventive/curative answers to these problems. Give citizens the right to health, increase budget allocation for health, and get competent, qualified people to head health departments and ministries. More importantly, have efficient science administrators and stop interfering in their work by recommending research grants to sycophants and blue-eyed boys.

***Ethicist:*** If you are an ethicist, you now know the task cut out for you. Just carry out a relentless campaign so researchers, policy planners, governments, science administrators, and the biomedical industry cannot deviate from ferreting out the truth about diseases of poverty and lifestyle. Accept no compromise to ethical conduct in researchers and industry. It's a perennial and unrelenting fight, and only those who have the guts and stamina to go on and on and on need to stick on here. Ethics is no part-time job, nor is it one that earns handsome perks. The work done well is often its only reward; and often the other reward is enemies all around who love to hate you, but know in their hearts that you are right after all.

***Man of religion:*** If you are a man of religion, you now know the task cut out for you. Your insight cannot but make it clear how mankind suffers from diseases due to defective lifestyles, economic/emotional deprivation, and lack of spiritual rootedness. You know what ensures well-being and leads to longevity. You know the benefits of simplicity. You need to get the man of science to listen to you and your insights. Stop pulling him down or his branch. He works under methodological constraints that you do not have to face. He needs your insights and he needs your patience to understand those insights; he needs time, and your persistence, to help him make experimental models out of your insights. But collaborate you must, for science combined with religion offers the greatest chance for complete human development and well-being. Nothing, just nothing, can match or derail this combination of science and religion if and when it comes about. Also, equally important, the followers of religion are driven by a strong faith, which is often blind. It becomes all the more important that such faith is never, ever manipulated. For then, religion cannot become the means for human development; it becomes, instead, the means for human exploitation; which, knowing the powerful hold it has on the human psyche, it can easily degrade into.

***Alternate/complementary medicine practitioner:*** If you are an alternate/complementary medicine person, you now know the task cut out for you. You are aware of lifestyle problems and have many insightful remedies to offer, which the person of mainstream medicine is sceptical of at present, and not without reason. Be more of complementary and less of alternate medicine. Mainstream medicine has a lot to offer, and it will serve the cause better if you complement their efforts rather than try to prove your alternative status. Moreover, stop going overboard with tall claims that only conceal an inner insecurity. Offer scientific proofs the way mainstream medicine demands and does, for they dictate the research agenda and procedures today—and not without justification. There are many therapies that promote well-being and longevity that you may have to offer, but they must stand the acid test of scientific verifiability and replication across geographical boundaries.

***Conscientious citizen:*** If you are a conscientious citizen of your country, you now know the task cut out for you. To read what is written as a challenge to all the guys above and not relent till they have done it. For they subsist on you, and can survive only till you allow them to. Simplify your life and get researchers to study how simplicity impacts health. Stop being taken for a ride by the empty promises of callous regimes and bureaucrats and ask what action is being taken to promote health, reduce hunger, eliminate poverty, and ensure longevity with well-being. Stop feeling lost when scientists give long-winded explanations, which are often only a substitute for action, and urge them to find mechanisms to reduce stress and the related lifestyle diseases. Do not get exploited by smart researchers and their henchmen who may use you like a guinea pig to try out unsubstantiated therapies without your informed consent. Stop feeling helpless when your call for action elicits no response from the powers that be. Knowledge is power, and when you know what you need to get done and why, these people have no option but to do it; you must rid yourself of the shackles that poverty (in the developing) and lifestyle stress (in the developed) place on your psyche. Unite and carry out concerted efforts with NGOs, national and international, to make your voice heard. With the help of the knowledge that organisations like the WHO and its related agencies now place at your disposal (and with the explosion of knowledge that the WWW supplies, if only you know how to use it to further your cause), there is no way that the lethargy and complacency of the powers that be will not be shaken. Thus aroused, the slow wheels of change will gather momentum. For the government and power machinery are like sleeping giants. Once awakened, they can do wonders. You must leave the indolent giants with no alternative but to work that wonder. Thus, you take the first firm steps to achieve the glory of human development, which is at once both your journey and your destination.

***Citizens of a world community:*** As citizens of a world community, know that the world has indeed shrunk; peoples and cultures are closer. So much that is good in far off geographical areas is now available to us because the world has coalesced and people and societies have opened up. Along with this comes the realisation that mankind exists at various stages of development. Those in the developed world have the means to make life meaningful but have often lost the meaning of life itself, while those in the developing world are fighting for life but often have the recipes to make life meaningful. This is especially true of a society like India, which is rapidly growing out of its underdeveloped status to become a developed society; it boasts of an ancient civilization and a philosophical outlook based on a robust mix of the temporal and the spiritual. There is a vibrant indigenous biomedicine and related disciplines (for example, Ayurveda, Yoga, etc.) as well as a burgeoning corpus of modern biomedical knowledge in active conversation with the rest of the world. India should be especially careful that while striving for economic development and scientific/biomedical advance, it holds fast to the values that have added meaning and purpose to life; values that the ancients bequeathed it with their experiential knowledge down the centuries. In fact, a rediscovery and careful sifting of such values to determine those relevant to the modern times is an important task before the thinking Indian of today. In so doing, he may be doing a signal service both to Indian society and, by extension, to most other societies that are escaping, or have escaped, the vicious grip of the diseases of poverty but are about to land, or have already landed, in the lap of lifestyle diseases.

How the means that the developed world has combines with the recipes to make them meaningful that the developing world has-that is the challenge ahead for mankind as it gropes its way out of poverty, disease, despair, alienation, anomie, and the ubiquitous all-devouring lifestyle stresses, and marches with halting steps towards well-being and the glory of human development.

## Concluding Remarks

1. Diseases of poverty afflict populations in the developing world, while lifestyle diseases grip those in the developed sections of society. At present, positive health and well-being appear a distant goal for both.
2. Poverty is not just income deprivation but capability deprivation as well; poverty also means optimism deprivation.
3. While life expectancy may have increased in the haves, and infant and maternal mortality reduced, it does not necessarily ensure that well-being results. If it is bacteria in their various forms that are the culprit in infectious diseases, it is poverty/deprivation in its various manifestations that is the culprit in poverty-related diseases, and it is stress in its various avatars that is the culprit in lifestyle diseases. It is as though poverty and stress have become the modern “bacteria” of developing and developed societies, respectively.
4. For those societies afflicted with diseases of poverty, of course, the prime concern is to escape the deadly grip of poverty-disease-helplessness. But, while so doing, they must be careful not to land in the lap of lifestyle diseases. For the haves, the need is to seek inner rootedness, to combat lifestyle diseases, to reduce professional burnouts, to ensure longevity with well-being, to simplify life, declutter homes, downsize ambitions, avoid excessive debts, over-consumption, over-connectedness, and over-exposure to negativity.
5. Studies on well-being, longevity and simplicity need the concerted attention of researchers and science administrators.
6. How the means that the developed have combine with the recipes to make them meaningful that the developing have-that is the challenge ahead for mankind. This is especially true of a society like India, which is rapidly growing out of its underdeveloped status to become a developed society of the near future. It is an ancient civilization with a philosophical outlook based on a robust mix of the temporal and the spiritual, with vibrant indigenous biomedical and related disciplines, for example, Ayurveda, Yoga etc, as well as a burgeoning modern biomedical knowledge corpus in active conversation with the rest of the world. It should be especially careful that, while it does not negate the fruits of economic development and scientific/biomedical advance that seem to beckon it in this century, it does not lose the values that have added meaning and purpose to life, which the ancients bequeathed it with their experiential knowledge down the centuries.

### Take Home Message

1. Positive health appears a distant goal both for the haves and the have-nots at present.
2. For societies afflicted with the diseases of poverty, the prime concern is to spin out of the deadly grip of poverty-disease-helplessness. But, while so doing, be careful not to land in the lap of lifestyle diseases. For the haves, the need is to combat lifestyle diseases by reducing stress, which is only possible by adopting health-promoting behaviours, reducing burnouts, making life simple, combating negative emotions, and seeking rootedness to values.
3. Human development lies in defining and achieving well-being with longevity. India, with its rich cultural heritage (which includes its indigenous biomedicine) and its new-found economic prosperity and scientific/ biomedical advance, may hold such a recipe. It must be careful not to abandon it in its surge towards economic development.

## Questions That This Paper Raises

1. How do we organise a mass movement to achieve positive health?
2. Is longevity with well-being an achievable target?
3. Is simplifying life likely to promote health at the cost of economic development?
4. How do we ensure that diseases of poverty reduce/disappear?
5. How do we combat lifestyle diseases?
6. Can we visualise a situation where the man of medicine will collaborate with the man of religion to ensure well-being?
7. How do we ensure achievement and success without promoting negative emotions?
8. Can we ensure lifestyle changes connected with diet, exercise, smoking, and drinking without ensuring lifestyle modifications that reduce hostility, antagonism and other negative emotions?
9. Can inner rootedness, inner peace and spirituality ever become the subject matter of large-scale scientific research?
10. How do we make the keys to longevity-moderation of lifestyle, care for physical self, self-sufficiency, and simplicity-the key determinants of our lifestyle, even as we do not abandon economic prosperity and competitiveness?
11. How do we establish cooperation and consensus, and not competition and ambition, as the key determinants of our personal and social consciousness?
12. Will mankind continue to pay the price-in the form of disease, wars, inter- and intra-personal stress, and exploitation and suppression of vulnerable populations-rather than realise that well-being lies in simplicity, cooperativeness, self-transcendence, thinking beyond individual human existence, character strengths and virtues (such as hope, compassion, and courage), letting go of all struggles, working in the service of others, and growing in self-awareness?

## About the Authors

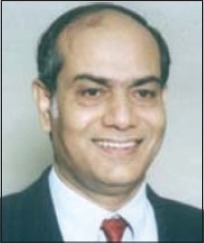



Ajai R. Singh, M.D., is a psychiatrist and Editor, Mens Sana Monographs, (http://www.msmonographs.org). He has written extensively on issues related to psychiatry, philosophy, bioethical issues, medicine, and the pharmaceutical industry.

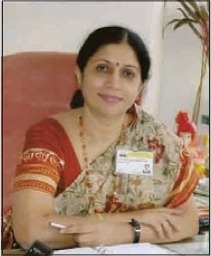



Shakuntala A. Singh, Ph.D., is Principal, Reader and Head, Department of Philosophy, K.G. Joshi College of Arts and N.G. Bedekar College of Commerce. She is also Deputy Editor of MSM. Her areas of interest are Indian Philosophy, Bio-ethics, Logic, and the Philosophy of Science.

## Footnotes

* It is interesting to note that there is a journal devoted to application of psychology to improve the quality of work life, the work environment, the individual, and the work.family interface: Journal of Occupational Health Psychology. See http://www.apa.org/journals/ocp/ for details. -Ed.
